# Transcriptional Regulation of Postnatal Cardiomyocyte Maturation and Regeneration

**DOI:** 10.3390/ijms22063288

**Published:** 2021-03-23

**Authors:** Stephanie L. Padula, Nivedhitha Velayutham, Katherine E. Yutzey

**Affiliations:** 1 The Heart Institute, Division of Molecular Cardiovascular Biology, Cincinnati Children’s Hospital Medical Center, Cincinnati, OH 45229, USA; stephanie.padula@cchmc.org (S.L.P.); nivedhitha.velayutham@cchmc.org (N.V.); 2 Molecular and Developmental Biology Graduate Program, Division of Developmental Biology, Cincinnati Children’s Hospital Medical Center, University of Cincinnati College of Medicine, Cincinnati, OH 45229, USA; 3 Department of Pediatrics, University of Cincinnati College of Medicine, Cincinnati, OH 45229, USA

**Keywords:** cardiomyocyte, transcription factors, nucleation, polyploidization, hypertrophy, sarcomere, mitochondria

## Abstract

During the postnatal period, mammalian cardiomyocytes undergo numerous maturational changes associated with increased cardiac function and output, including hypertrophic growth, cell cycle exit, sarcomeric protein isoform switching, and mitochondrial maturation. These changes come at the expense of loss of regenerative capacity of the heart, contributing to heart failure after cardiac injury in adults. While most studies focus on the transcriptional regulation of embryonic or adult cardiomyocytes, the transcriptional changes that occur during the postnatal period are relatively unknown. In this review, we focus on the transcriptional regulators responsible for these aspects of cardiomyocyte maturation during the postnatal period in mammals. By specifically highlighting this transitional period, we draw attention to critical processes in cardiomyocyte maturation with potential therapeutic implications in cardiovascular disease.

## 1. Introduction

The adult mammalian heart lacks regenerative capacity, which is a contributing factor in heart failure after myocardial injury. However, neonatal cardiomyocytes can proliferate and promote regenerative cardiac repair following injury in rodents and swine [1,2,3]. In some vertebrates, such as newts and zebrafish, there is a capacity for cardiac regenerative repair and cardiomyocyte proliferation throughout life [4]. However, while mammalian fetal cardiomyocytes proliferate during development, the vast majority of adult cardiomyocytes are mitotically quiescent [5,6]. Even following injury, cell cycle activity in adult cardiomyocytes is limited to increased multinucleation and polyploidy without cytokinesis [7,8]. In swine, while cell cycle arrest does not coincide with loss of regenerative potential, cardiomyocyte cytokinetic mechanisms are repressed approximately one week after birth with the onset of multinucleation [2,3,9]. These cardiomyocyte maturational dynamics, which occur in the first few weeks after birth in small and large mammal model systems, may be prolonged in humans to the first few years before puberty, although this is controversial [10,11]. Together, these cellular processes of cardiomyocyte mitotic arrest and inability to complete cell division are concurrent with the loss of regenerative capacity following cardiac injury in humans and other mammals.

After birth, the mammalian heart undergoes dramatic changes in size, oxidative capacity, and energy production due to the increased cardiac demand [12,13,14,15,16,17,18]. At the same time, cardiomyocytes respond with adaptations in calcium excitation-contraction coupling, ATP production, and contractile function related to sarcomeric protein isoform expression and hypertrophic growth. Despite having similar contractile functions, fetal and adult mammalian cardiomyocytes differ on molecular, biochemical, morphological, and structural levels. While these cardiomyocyte maturation events coincide with loss of regenerative capacity in juvenile and adult rodent cardiomyocytes, the specific molecular and cellular bases of the inability to regenerate have not yet been identified.

Recent studies have identified critical transcriptional regulators of gene expression related to cardiomyocyte maturation in neonatal rodents [19]. However, despite the clinical relevance for understanding how these transcriptional regulators contribute to cardiomyocyte maturation, most studies focus on their function in cardiomyocytes either during embryonic development or during adulthood, with less information available related to the postnatal period. Here, we discuss cellular and molecular changes that occur in cardiomyocytes during the postnatal transition from fetal to adult stages and review recent progress on transcriptional regulation of this maturation process. Since understanding the dynamic transcriptional regulation of cardiomyocyte maturation in the postnatal period may help elucidate new targets for cardiac therapy, this review will also highlight areas requiring further investigation.

## 2. Overview of Postnatal Cardiomyocyte Maturation

During the postnatal period, cardiomyocytes in mammals undergo maturational transitions that lead to the adult cardiomyocyte phenotype. Here, we discuss characteristic hallmarks of mature cardiomyocytes, including mitotic arrest, multinucleation/polyploidization, growth by hypertrophy, transition to oxidative metabolism, and expression of mature sarcomeric contractile protein isoforms. Numerous genes involved in these processes are direct downstream targets of major transcription factors that control cardiomyocyte maturation states, which will be discussed in detail later in this review.

### 2.1. Cell Cycle Arrest and Multinucleation

**Figure 1:**
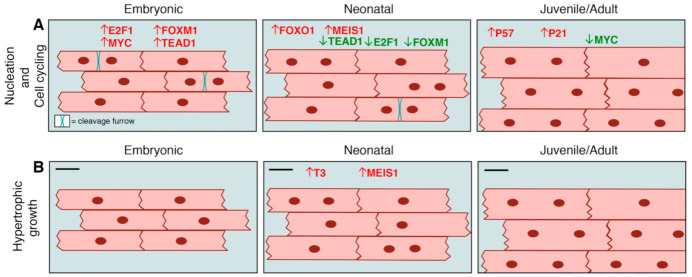
Transcriptional control of nucleation, cell cycling, and hypertrophic growth in embryonic, neonatal, and juvenile/adult rodent cardiomyocytes. (**A**) Embryonic/fetal cardiomyocytes are primarily mononucleated and proliferate to drive cardiac growth prenatally due to high levels (red) of Tead1, E2f1, Foxm1, Myc. After birth, karyokinesis in the absence of cytokinesis increases as cardiomyocytes mature and become multinucleated. This is concomitant with the downregulation (green) of cell cycle promoting factors such as Tead1, E2f1, Foxm1, Myc; together with the upregulation (red) of cell cycle inhibitory factors such as Foxo1, Meis1, p57, p21. (**B**) Hypertrophic growth increases after birth with the induction of multinucleation. This is associated with increased levels of T3 thyroid hormone and Meis1 in adult cardiomyocytes. Refer to text for citations.

In mammals, evidence suggests that the predominantly mononucleated and diploid state of embryonic/fetal cardiomyocytes is essential for the high proliferation rates observed in these hearts [13,20,21,22,23]. During postnatal cardiomyocyte cell cycle arrest, karyokinesis (nuclear division) in the absence of cytokinesis (cell division), also known as endoreplication, results in predominantly binucleated cardiomyocytes in a post-mitotic quiescent state within 10 days after birth in rodents (Figure 1A) [13,21,24,25]. By contrast, in pigs, while cytokinetic arrest occurs within the first weeks after birth, karyokinesis in the absence of cytokinesis (endoreplication) continues beyond two postnatal months, resulting in extensive bi- and multi-nucleation, with up to 32 nuclei per individual cardiomyocyte [9]. Similarly, in humans, nuclear polyploidization and binucleation of cardiomyocytes increases within the first few years after birth [6,10]. Conversely, the lifelong capacity for cardiac regeneration in adult zebrafish hearts has been linked to the large population of mononucleated-diploid cardiomyocytes capable of dedifferentiation and proliferation [26]. Direct perturbation of cytokinesis in proliferating cardiomyocytes leads to loss of regenerative potential in both zebrafish and neonatal mice, supporting a causative role [13,26]. Cardiomyocyte polyploidization also contributes to the injury response in adult cardiomyocytes, which undergo maladaptive multinucleation or increased nuclear polyploidization in disease/injury conditions [22]. Thus, cardiomyocyte polyploidization may be both a consequence of, and directly responsible for, the transition from a fetal-like proliferative state to a post-mitotic terminally-mature state.

### 2.2. Switch to Hypertrophic Cardiomyocyte Growth

Fetal cardiomyocytes exhibit hyperplastic growth, i.e., growth by proliferation. However, with cardiomyocyte cell cycle arrest and loss of proliferative capacity, cardiomyocytes switch from hyperplastic to a hypertrophic (increase in cell size) mode of growth, within the first two weeks after birth in rodents [23] (Figure 1B). Conventionally, this increase in cardiomyocyte size occurs diametrically, measured by increased cell cross-sectional area or cell width. In zebrafish, there is no transition to hypertrophic cardiomyocyte expansion, as proliferative cardiomyocytes remain embryonic-like with minimal cytoplasmic area and large central nuclei throughout life [27]. In swine, cardiomyocyte cell length increases with multinucleation in the months after birth, and hypertrophic growth, as indicated by increased cell diameter, is apparent two to six months after birth. Notably, rapid cardiomyocyte growth is achieved in swine through elongation and multinucleation, with up to 16 nuclei per cardiomyocyte seen at six months after birth [9]. Thus, in mammalian species, postnatal cardiomyocyte terminal maturation involves increases in cardiomyocyte cell size with variable numbers of nuclei during the juvenile-to-young adult stages of development.

### 2.3. Transition to Oxidative Metabolism

Fetal cardiomyocyte energy production occurs primarily by glycolysis in rodents, although there is evidence of mid-to-late gestational onset of oxidative metabolic pathways in some large mammals, such as sheep [28]. In zebrafish hearts, increased glycolysis and pyruvate metabolism is noted in cardiomyocyte proliferation and regenerative repair of the heart [29]. In rodent hearts, cardiomyocyte metabolism undergoes rapid transition from glycolytic to fatty acid oxidation in the first few days after birth with postnatal increase in oxygen consumption [30]. Increased hypoxia and decreased reactive oxygen species (ROS) in cardiomyocytes also have been shown to promote cardiomyocyte proliferation and regenerative repair of the heart after injury [31,32].

**Figure 2:**
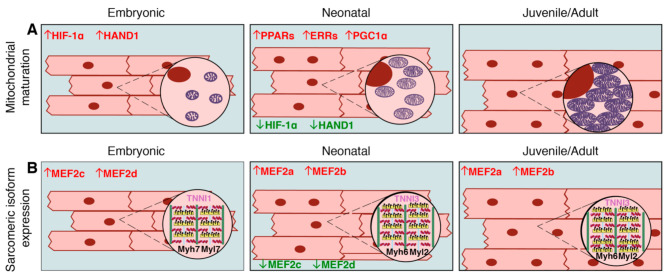
Transcriptional control of mitochondrial maturation and sarcomeric protein isoform expression in embryonic, neonatal, and juvenile/adult rodent cardiomyocytes. (**A**) As cardiomyocytes mature, mitochondria number and size increase, as does the number of cristae. These maturational changes are associated with downregulation (green) of embryonic transcription factors including Hif-1α and Hand1, and upregulation (red) of neonatal/adult transcription factors including PPARs, ERRs, and PGC1α. (**B**) Embryonic cardiomyocytes express immature Tnni1, Myh7, and Myl7, which are replaced by Tnni3, Myh6 and Myl2 during the postnatal period. Sarcomere number also increases during postnatal maturation and hypertrophic growth. This isoform switching during maturation is associated with downregulation of transcription factors, such as Mef2c and Mef2d, and upregulation of Mef2a and Mef2b. Refer to text for citations.

Due to increased energy demands, cardiomyocyte mitochondria undergo maturation characterized by high rates of mitochondrial biogenesis and size increase, organization of cristae, and broadened localization across cellular compartments, which is in contrast their perinuclear localization in early embryonic hearts [33] (Figure 2A). Together, this remodeling is necessary for adequate ATP production in adult cardiomyocytes [34,35]. Human and pig mitochondrial dynamics during heart development are not well-characterized, but, given the shared atmospheric oxygen environment between these species and rodents, along with the incidence of human neonatal mitochondrial cardiomyopathies associated with oxidative phosphorylation defects [36,37,38], it is likely that a similar mitochondrial maturation occurs in large mammal cardiomyocytes. In the regenerative adult zebrafish model, the state of the mitochondria more closely resembles that of the neonatal mouse, with increased glycolysis and pyruvate metabolism noted in proliferative cardiomyocytes and regenerative repair of the heart [29]. These data support a link between the extent of mitochondrial maturation and cardiomyocyte proliferative capacity in the heart.

### 2.4. Fetal to Adult Contractile Protein Isoform Switching

Cardiomyocytes are comprised of functional contractile units known as sarcomeres, which consist of actin-rich thin filaments, myosin-rich thick filaments, titin, and their associated proteins [39]. Together, they form the contractile apparatus necessary for generating cardiac output. Sarcomeres form early in heart development with the initiation of cardiac function, and sarcomeric maturation continues with increased force production in the neonatal period [40]. In embryonic and early neonatal stages in rodents, fetal isoforms of sarcomeric contractile proteins, such as Myh7 (β-myosin heavy chain), Tnni1 (slow skeletal muscle troponin I), and Myl7 (myosin light chain 7), predominate in the heart. During postnatal cardiomyocyte maturation in rodents, sarcomeres undergo switching to adult isoforms, including Myh6 (α-myosin heavy chain), Tnni3 (cardiac troponin I3), and Myl2 (myosin light chain 2) in the ventricles (Figure 2B) [41,42]. In contrast, the regenerative adult zebrafish express *vmhcl* and *myl7*, orthologous to fetal murine *Myh7* and *Myl7*. Further, adult zebrafish express *tnnt1* (troponin T1) and *tnnt2*, which are orthologous to mouse *cTnT1* and *cTnT2* (cardiac troponin T 1/2) respectively [43]. Zebrafish do undergo some sarcomeric isoform switching during early embryogenesis- namely, switching from *tnnt3a* to *tnnt3b*, and alternative splicing of *tnnt2*. However, these switches occur within 72 h post-fertilization, and there are no established isoform differences between embryonic zebrafish and adult zebrafish [44]. A direct correlation between zebrafish and mammalian isoforms may not be exact, as sarcomeric isoform maturational dynamics in zebrafish are not well-characterized, and the proteins are not fully conserved with mammals. Nonetheless, the loss of embryonic sarcomeric isoform expression in postnatal quiescent rodent cardiomyocytes, compared to the retention of embryonic isoforms in regenerative adult zebrafish cardiomyocytes, demonstrates a potential link between sarcomeric isoform expression and proliferative capacity.

## 3. Transcriptional Regulation of Postnatal Cardiomyocyte Maturation

Multiple transcription factors have critical roles in regulating specific stages of cardiomyocyte maturation, such as mitotic cell cycling, regulation of karyokinesis and cytokinesis, hypertrophic growth, adult sarcomeric contractile protein gene expression, fatty acid metabolism, and mitochondrial biogenesis and maturation. Despite their importance, we lack a full understanding of the expression patterns, binding partners, and downstream mechanisms of these transcriptional regulators during postnatal cardiomyocyte maturation. Here we review transcriptional regulation of cardiomyocyte maturation in the postnatal mammalian heart, with parallels drawn to other vertebrate model systems, and focus on the relationship between cardiomyocyte maturation and proliferative capacity. The major transcriptional regulators discussed are summarized in Section 4.

### 3.1. Transcriptional Regulation of Prenatal Versus Postnatal Cardiomyocyte Cell Cycling

Prior to birth, proliferation of the newly-differentiated myocytes in the developing mammalian heart depends on the activity of multiple transcription factors [45,46,47]. A major change governing cardiomyocyte maturation is cell cycle arrest which is accompanied by disassembly of nuclear centrosomes [48]. This cellular process contributes to the loss of cardiomyocyte cytokinetic capacity and increased nucleation/polyploidization implicated in cardiomyocyte maturation, concomitant with the loss of regenerative healing of the rodent heart following injury [7,8]. Examination of the transcriptional regulation of the transition from proliferative mitotic activity (karyokinesis followed by cytokinesis) versus polyploidizing mitotic activity (karyokinesis with no cytokinesis) in the postnatal period thus is relevant to understanding the loss of regenerative capacity.

Some of the major pathways for cardiomyocyte proliferation in embryonic cardiomyocytes have decreased activity during the postnatal period thus contributing to cell cycle arrest as determined in rodents. For example, the Hippo effector Yap1 promotes embryonic cardiomyocyte proliferation in combination with its co-factor, Transcriptional enhancer factor Tef-1 (also known as Tead1) [49,50,51]. Tead1 co-binding with Yap1 is required for proliferation in the perinatal period, as demonstrated by reduced proliferation and cardiomyopathy in mice with conditional loss of Tead1 in cardiomyocytes driven by αMHC*-Cre* (α-Myosin Heavy Chain) [52]. In contrast, decreased Hippo-Yap signaling and downregulation or sequestration of its nuclear target Tead1 in postnatal murine hearts is required for cardiomyocyte cell cycle arrest [51] (Figure 1A). Another major developmental pathway, Neuregulin (NRG) signaling through ERB receptors, is also necessary for cardiomyocyte proliferation in the embryonic rodent heart [53,54]. Decreased NRG signaling and reduced ErbB expression during postnatal maturation in mouse hearts is necessary for cardiomyocyte cell cycle arrest [55], with premature ErbB4 deletion in neonatal mice leading to reduced perinatal proliferation [56]. Conversely, constitutive activation of Yap1 or ErbB leads to prolonged unrestrained cardiomyocyte proliferative activity after birth resulting in cardiomegaly and eventually heart failure.

Developmental cardiac transcription factors, including T-box transcription factors Tbx5 and Tbx20, also contribute to embryonic cardiomyocyte proliferation. Tbx5 is required for embryonic heart chamber growth, and mice conditionally lacking Tbx5 in embryonic cardiomyocytes exhibit hypoplastic ventricles associated with downregulation of cell cycle genes and reduced proliferation [57]. Tbx5 also interacts with the transcription factor Gata4 to promote the activation of cyclin dependent kinases such as *Cdk4* and *Cdk2* during embryonic heart development [58]. Although Tbx5 is expressed in the postnatal period, the major phenotype of adult mouse cardiomyocytes lacking Tbx5 is atrial fibrillation associated with downregulation of its ion channel target genes *Nppa*, *Gja5*, and *Scn5a* [59], suggesting that its role switches from predominately regulating proliferation in embryos to regulating conduction or homeostasis postnatally. Similarly, embryonic mouse cardiomyocytes lacking Tbx20 arrest in the G_1_-S phase of the cell cycle leading to embryonic lethality [60,61,62,63]. Chromatin immunoprecipitation (ChIP)-Seq experiments for Tbx20 targets in E11.5 mouse cardiomyocytes reveal direct binding of Tbx20 to the promoter regions of *Ccna2*, *Cdc6*, *Mycn*, and *Erbb2* to promote their transcription [64]. Tbx20 is also expressed in adult cardiomyocytes, albeit at a reduced level compared to fetal cardiomyocytes, where it directly represses cell cycle inhibitor genes *Cdkn1a*, *Meis1*, and *Btg2* [65,66]. It is unknown if interacting cofactors facilitate this switch in Tbx20 target binding and repressor versus activator function. Likewise, Gata4 has been implicated in regulation of cardiomyocyte proliferation during prenatal development but promotes cardiac hypertrophic growth after birth [67,68]. Thus, multiple developmental transcription factors switch from a proliferative to maturational role in cardiomyocytes postnatally, although the underlying mechanisms remain poorly understood.

Transcription factors and signaling pathways that are critical for embryonic cardiomyocyte cell cycle activity are downregulated or have reduced activity during the postnatal period, thus permitting cardiomyocyte cell cycle exit [47]. For example, AKT-mediated nuclear localization of the forkhead box transcription factor FoxM1 promotes cardiomyocyte proliferation in the developing heart [69], but FoxM1 expression decreases during the postnatal period [19] (Figure 1A). Likewise, the transcription factors E2f2 and E2f4 promote embryonic cardiomyocyte mitotic activity and are downregulated postnatally (Figure 1A); however, forced expression of E2f2/4 in adult cardiomyocytes leads to massive cell death [70,71,72]. Finally, Isl1 promotes embryonic cardiomyocyte proliferation by activating Fgfs and Bmps [73], which elicit a downstream proliferative response, and cooperate with Gata4 to express Hand2, another transcription factor that activates proliferative genes [74,75]. Recent evidence suggests that Isl1 promotes this response by interacting with the Brg1-SWI/SNF chromatin remodeling complex, which is only expressed prior to the postnatal period [76]. Since the precise mechanism of cardiomyocyte karyokinesis versus cytokinesis is not yet elucidated, it is unclear which specific process these transcriptional regulators direct in embryonic and fetal cardiomyocytes. As such, although these represent potential targets for reactivation of cell cycle activity postnatally, their overexpression may yield multinucleation or polyploidization rather than true cellular proliferation.

Major transcriptional regulators that actively promote cardiomyocyte cell cycle exit during the week after birth in rodents include FoxO1 and Meis1 (Figure 1A). Activation and nuclear localization of FoxO1 increases during the postnatal period in mice, concomitant with direct induction of its target cyclin-dependent kinase (CDK) inhibitor genes *p21* and *p27*, which promote cell cycle arrest [69]. Meis1 is a homeobox containing transcription factor with increased expression postnatally that inhibits cardiomyocyte cell cycling. Meis1 overexpression in neonatal mouse cardiomyocytes causes premature cell cycle exit by activating its CDK inhibitor target genes *p15*, *p16*, and *p21* [77]. Finally, downregulation of the T-box transcription factor Tbx20 during the postnatal period coincides with increased expression cell cycle inhibitory transcription co-factors such as Btg2 in postnatal cardiomyocyte cell cycle exit [65]. Thus, multiple transcriptional regulators directly influence loss of proliferative activity in early postnatal cardiomyocytes.

Cardiomyocyte proliferative and developmental transcriptional regulators expressed in embryos and neonates are attractive candidates to promote proliferative and regenerative repair responses in adult cardiomyocytes. Recent efforts to promote proliferation in adult cardiomyocytes by overexpressing transcriptional regulators that have critical roles in cardiomyocyte proliferation and maturation have been met with challenges. For example, Tbx20 overexpression (Tbx20^OE^) in fetal mouse cardiomyocytes using the β-Myosin Heavy Chain *Cre* (*βMHC-Cre*) line or in adult mouse cardiomyocytes with tamoxifen induction using the α-Myosin Heavy Chain MerCreMer (α*MHCMCM*) line results in persistent, non-pathological proliferation into adulthood, associated with the upregulation of cell cycle genes *Ccnd1*, *Ccne1*, and *Igf1*, and the downregulation of cell cycle inhibitory genes *Cdkn1a*, *Meis1*, and *Btg2*, which may contribute to improved survival and function after MI [65,78]. Perplexingly, although *βMHC-Cre-*induced or adult α*-MHCMCM* tamoxifen induced Tbx20^OE^ results in proliferation without hypertrophy or reduced cardiac function [65,78], overexpression of Tbx20 in mice driven by the α-MHC promoter causes enlarged hearts with abnormal ventricular structure and poor cardiac function, suggesting that timing or dosage of Tbx20 affects its pro-proliferative activities [79].

Similarly, Yap1^OE^ has different phenotypes in adult cardiomyocytes depending on mode of overexpression. For example, reactivation of Hippo-Yap signaling through expression of constitutively nuclear Yap promotes cardiac regeneration in postnatal rodent hearts [80,81], and AAV9-mediated Yap1^OE^ results in proliferation without hypertrophy and improved outcome after MI [82]. However, a constitutively active version of Yap (YAP5SA) causes hyperplasia in adult cardiomyocytes and death, despite activation of cell cycle-related genes and the proliferation-inducing transcription factors Myc, E2f1 and E2f2 [83]. In either Tbx20^OE^ or Yap1^OE^, it is unclear whether the dosage of transcription factor causes differential responses, or whether there are more complex regulatory mechanisms at play depending on the timing of overexpression. Future studies should elucidate these possibilities. While ectopic ErbB2 expression is sufficient to trigger heart regenerative repair via cardiomyocyte proliferation and dedifferentiation [55], it has also been linked with cardiomegaly with long-term expression. Interestingly, transient over expression of ErbB2 or Yap1 leads to increased cardiomyocyte proliferation, decreased scarring, and improved cardiac function after MI [55]. Further, recent studies highlight crosstalk between ErbB2/Yap1-mediated cardiac regenerative mechanisms, where Yap activation occurs downstream of cytoskeletal alterations by ErbB2^OE^, via mechanotransduction signaling [84]. Depletion of Meis1 and FoxO1/O3, which normally function as inhibitors of cell cycle progression in postnatal cardiomyocytes, results in increased cardiomyocyte proliferative response limited to one additional cell cycle [69,77]. In contrast, overexpression of the cell cycle inhibitory transcription factor Meis1 prevents appropriate regeneration in neonatal mouse hearts after MI, associated with premature cell cycle exit and hypertrophic growth of cardiomyocytes [77]. While these studies support the possibility that adult mammalian cardiomyocytes are capable of regeneration, they have also highlighted the complexity underlying cardiomyocyte proliferation. Studies delineating the effects of specific timing and dosage of overexpression may be needed to achieve regeneration without heart pathology.

A major roadblock to induction of cardiomyocyte proliferation is the ability to manipulate specific regulatory mechanisms that control karyokinesis and cytokinesis, which are responsible for generation of new cardiomyocytes through proliferation, versus multinucleation and nuclear polyploidization in postnatal cardiomyocytes [7]. Some “fetal-reversion” studies based on developmental transcription factor overexpression have demonstrated successful induction of increased numbers of mononucleated cardiomyocytes concomitant with induction of cardiomyocyte proliferative capacity [65,78,82,83]. However, it is unclear whether forced cell cycle activity promotes cytokinesis, or whether certain transcription factors can also directly target genes relevant to multinucleation during postnatal cardiomyocyte maturation. A recent scRNA-seq experiment performed on adult mouse cardiomyocytes revealed very few transcriptional differences between mono- and binucleated cells [85]. This study was limited to only considering a certain size and shape of cardiomyocytes, and thus may have failed to fully capture transcriptional changes associated with binucleation. However, this finding is supported by a recent study determining that ploidy and nucleation do not contribute to the injury response of adult mouse cardiomyocytes after myocardial infarction [86]. Nonetheless, additional studies distinguishing these possibilities will reveal numerous insights into the regulatory mechanisms at work in postnatal cardiomyocytes.

### 3.2. Transcriptional Regulation of the Postnatal Induction of Hypertrophic Growth in Cardiomyocytes

Transcription factors that govern the switch from hyperplastic to hypertrophic growth of cardiomyocytes during the postnatal period are not well-defined. However, in addition to regulating cardiomyocyte cell cycling, many developmental transcription factors also likely have a role in regulating postnatal transition to hypertrophic cardiomyocyte growth upon cardiomyocyte cell cycle arrest, as well as pathological hypertrophy in disease conditions. For example, deletion of Gata4, which is indispensable for proliferation in the developing embryonic cardiomyocytes, reduces hypertrophy due to pressure overload in adult cardiomyocytes, indicating that it may induce normal hypertrophic growth in the postnatal period [68,87,88]. Indeed, a recent RNA-Seq and ATAC-Seq analysis of P1, P14, and P56 mouse cardiomyocytes reveals upregulation of Gata transcriptional targets from P1 to P56 [19]. However, by deleting Gata4, cardiac function declines and heart failure occurs rapidly, indicating that a proper dosage of Gata4 may be the key to modulating hypertrophy in the postnatal period. Another transcription factor implicated in this process is Nkx2.5, also a fundamental cardiac developmental transcription factor [89]. Nkx2.5 interacts with Gata4 to synergistically activate their transcriptional targets, such as B-type natriuretic peptide (BNP), so it is unsurprising that Nkx2.5 likewise induces hypertrophy in adult cardiomyocytes upon deletion [90,91]. What governs the loss of proliferative function of Gata4 and Nkx2.5 in hypertrophic growth during the postnatal period and later in disease is unclear but represents an interesting avenue for future mechanistic studies. Interestingly, adenovirus-mediated overexpression of Gata4 at P7 results in improved cardiac function after myocardial cryoinjury, accompanied with increased proliferation, no indications of hypertrophy, and reduced scar size [92]. This study demonstrates the delicate balance required to promote hyperplastic growth rather than hypertrophic growth in postnatal cardiomyocytes. Exploring the role of these various candidate developmental and neonatal transcription factors that are involved in hyperplastic versus hypertrophic growth in postnatal cardiomyocytes may help discover potential therapeutics for humans after MI.

Other major regulators of cardiomyocyte hypertrophy include signaling via Tri-iodo-l-thyronine (T(3)) hormone and calcineurin. T3 promotes cell cycle exit and binucleation along with expansion of cardiomyocyte size in fetal sheep [93,94] (Figure 1B). Mice lacking thyroid hormone receptors, but with intact thyroid hormone levels, undergo contractile abnormalities associated with decreased calcium handling in cardiomyocytes [95,96,97]. In addition, FoxO1 regulates type II iodothyronine deiodinases (Dio2) to promote postnatal induction of cardiomyocyte hypertrophy in response to Thyroid Hormone [98]. Finally, recent studies in mice demonstrate that the calcium-activated protein phosphatase calcineurin is activated postnatally to promote nuclear localization of Hoxb13, a co-factor of Meis1, and drive hypertrophic growth (Figure 1B). Indeed, the combined deletion of both Hoxb13 and Meis1 decreases postnatal cardiomyocyte hypertrophy, while promoting robust, non-pathological proliferation via multiple cell cycle targets including *Cdkn1a*, *Cdkn1b*, and *Tead1* [99]. These studies indicate that the postnatal cardiomyocyte cellular environment, subject to hormone levels and downstream signaling, contributes to increased hypertrophic growth of cardiomyocytes after birth.

### 3.3. Transcriptional Regulation of Fetal and Adult Sarcomeric Isoform Gene Expression

The Mef2 family of transcription factors has conserved functions in regulating sarcomeric protein gene expression both in zebrafish and mice. Several Mef2 isoforms exist, primarily controlled by alternative splicing, each with a critical role in cardiac development. Zebrafish *mef2c/d* knockdown embryos fail to express myosin heavy chain genes [100]. Mice express four isoforms of Mef2- Mef2a, Mef2b, Mef2c, and Mef2d. Of these, Mef2a and Mef2b are the most abundantly expressed isoforms postnatally [101] (Figure 2B). Studies in mice demonstrate that Mef2 proteins activate *Myh6* expression while physically interacting with Gata4 [102], while Tbx5 and Mef2C physically interact to promote synergistic expression of *Myh6* [103]. Interestingly, most Mef2a-deficient neonates die between P5 and P10 with fragmented myofibrils, but no dysregulation of myosin heavy chain genes *Myh6* or *Myh7* [104]. This may be explained by redundancy between Mef2a and Mef2d. In the same study, a Mef2*-lacZ* binding site reporter is active in cardiomyocytes depleted of Mef2a, indicating that Mef2d shares some binding targets with Mef2a. Furthermore, overexpression of Mef2a, Mef2c, or Mef2d in postnatal mouse cardiomyocytes results in abnormal myocardial growth and sarcomere disorganization [105,106,107,108]. Yet, it is unclear how these Mef2 isoforms, which are both spatially and temporally regulated, contribute individually or redundantly to postnatal sarcomere maturation.

Other transcription factors implicated in regulation of postnatal sarcomeric gene expression are Gata4, Nkx2.5, and Tbx20. Gata4 and its transcriptional coactivator Ankrd1 (Ankyrin Repeat Domain 1) promote *Myh6* expression in neonatal rat cardiomyocytes. In addition, Ankrd1 knockdown in rat cardiomyocytes prevents growth during the postnatal period [109]. Nkx2.5 overexpression in postnatal mice, using the αMHC (*Myh6*) promoter, promotes myofibrillar disorganization [110]. However, this phenotype lies downstream of miR-1 activation by Nkx2.5, which may mean that Nkx2.5 indirectly regulates sarcomere organization. Finally, CHIP-Seq in adult, but not embryonic, mouse cardiomyocytes, reveals Tbx20 binding to genes related to sarcomere and myofibrillar organization [64,66]. This is in accordance with Tbx20′s differential role in fetal versus adult cardiomyocytes. Of particular interest is the binding of Tbx20 within the promoter region of Mef2 genes, suggesting that Tbx20 indirectly regulates sarcomeric protein expression upstream of Mef2. Whether Tbx20 differentially regulates Mef2 isoform expression throughout development is not known.

### 3.4. Transcription Factor Regulation of Mitochondrial Maturation in Cardiomyocytes

There is increasing evidence that transcriptional regulation of metabolic transitions is critical for cardiomyocyte terminal maturation together with loss of proliferation and regenerative capacity in adult mammals. Perhaps the most well-studied transcriptional regulators of mitochondrial function and homeostasis are the nuclear peroxisome proliferator-activated receptors (PPARs). The PPAR receptors that promote fatty acid oxidation (PPARα and PPARδ), along with their transcriptional coactivator PGC1α, are abundant in the postnatal heart [111,112,113] (Figure 2A). PPARα overexpression in mice results, not only in increased oxidation, but also in decreased glycolysis, while conditional loss of PPARα in cardiomyocytes results in decreased mitochondrial oxidative metabolism [114,115,116,117]. In cardiomyocytes, PGC1α also functions as a coactivator of estrogen-related receptors (ERRα and ERRγ), which are also important transcriptional regulators of mitochondrial oxidative metabolism [118,119,120,121] (Figure 2A). Consequently, ERRγ-null mice die within the first week of birth due to severe mitochondrial defects, and ERRα-null mice exhibit worse outcomes following cardiac pressure overload [118,120]. Transcriptional targets regulated by PGC1α in combination with either ERRs or PPARs consist of mitochondrial oxidative metabolism-related genes, such as succinate dehydrogenase subunits, electron-transferring flavoproteins, and components of oxidative phosphorylation and the electron transport chain (including *Atp5g3*, *Coq7*, *Cox6c*, *Ndufa8*, *Ckmt2*, and *Slc25a4*) [122].

Hif-1α activity underlies mitochondrial maturation during the shift from a hypoxic environment in fetal cardiomyocytes to a more oxygen-rich environment postnatally (Figure 2A). Hif-1α activity decreases immediately after birth and contributes to mitochondrial biogenesis and growth. In a mouse model with constitutively active Hif signaling, mitochondria remain immature postnatally, corresponding with low levels of the Hif-1α targets *Mfn1*, *Mfn2*, and *Opa1* [123]. Inducing Hif-1 activity in adult mice after MI promotes a robust regenerative response via engaging existing cardiomyocytes in proliferation [32], supporting a link between mitochondrial energy production and cell cycle activity in cardiomyocytes. Likewise, postnatal cell cycle activity depends upon high ATP production, and inhibition of ATP synthesis by oligomycin reduces the proliferative capacity of P1 mouse cardiomyocytes [124]. Hypoxia-dependent gene expression also involves activation of the transcription factor Hand1, which promotes glycolysis in fetal cardiomyocytes, but is downregulated postnatally (Figure 2A). Consequently, Hand1 overexpression in postnatal cardiomyocytes decreases ATP production, and surprisingly, improves outcome after MI, possibly by lowering ROS levels [125]. Increased hypoxia and decreased ROS in mouse cardiomyocytes has been reported to promote cardiomyocyte proliferation and regenerative repair of the heart after injury [32], which could be due to the ability of ROS to promote cell cycle exit and DNA damage in postnatal cardiomyocytes [31].

As the mitochondria of postnatal cardiomyocytes begin the large-scale production of ATP, they inevitably also produce significant amounts of ROS. Although healthy adult cardiomyocytes compensate for increased ROS via catalysis with a number of redox enzymes, ischemic hearts tend to produce excessive ROS. Under such conditions, the transcription factor Nrf2 forms a heterodimer with small Maf proteins, including MafF, MafG, and MafK [126,127]. These heterodimers activate enhancer elements known as antioxidant response elements (AREs), which promote the expression of genes coding for anti-oxidant enzymes, such as Gsta1 (Glutathione S-Transferase Alpha-1) and HO-1 (Heme Oxygenase 1) [128]. Nrf2 also induces the expression of the transcription factor Nrf1, which binds with its co-activator PGC1α to promote mitochondrial biogenesis both under normal developmental conditions and conditions of oxidative stress [129]. Interestingly, although Nrf2 is ubiquitously expressed, the protein is rapidly degraded via an ubiquitin proteasome-mediated pathway under non-stressed conditions [129]. Increased hypoxia and decreased ROS in mouse cardiomyocytes also promotes cardiomyocyte proliferation and regenerative repair of the heart after injury [32]. Thus, strategies aimed at promoting Nrf2 degradation after MI may force cardiomyocytes toward an immature mitochondrial state with decreased ROS production and a favorable environment for an improved regenerative response.

Overexpression or knockout studies in mice revealed that transcriptional regulation by the HIPPO pathway may contribute to mitochondrial maturation in addition to proliferation in cardiomyocytes. For example, overexpression of the Hippo effector Yap1 after MI promotes downregulation of genes involved in oxidative phosphorylation and metabolic processes [82]. However, it is unclear whether Yap1 directly influences mitochondrial maturation or promotes a fetal reversion phenotype in adult cardiomyocytes following MI, thus creating the proper environment for an immature mitochondrial state. Other studies have demonstrated that Yap1 promotes mitochondrial homeostasis by activating Parkin, a component of the outer mitochondrial membrane [130]. Yap1 also can interact with FoxO1 to promote survival following ischemic injury via stimulation of transcription of antioxidant genes [131]. In addition, adult mouse cardiomyocytes conditionally lacking Tead1, facilitated by *Myh6-Cre*, exhibit impaired oxidative phosphorylation and mitochondrial function [132]. Thus, there may be a direct influence of the HIPPO pathway on mitochondrial maturation in cardiomyocytes, which should be explored in greater detail.

Lastly, during cardiomyocyte maturation, isoform-switching occurs in expression of glycolytic and oxidative metabolism-related enzyme genes. For example, the fatty acid-binding protein Fapb3 is abundant in neonatal mice, but gradually decreases within the first three weeks after birth, compared to Fabp4 which is very low at birth and increases by P21 [41]. Interestingly, abnormally high levels of Fabp4 have been associated with heart failure due to increased transport of fatty acids into cardiomyocytes. Another isoform switch during mitochondrial maturation is the transition from Hexokinase1 (Hk1) in embryonic and neonatal rodent cardiomyocytes to Hexokinase2 (Hk2) in adult rodent cardiomyocytes. Overexpression of the embryonic isoform Hk1 in adult rat cardiomyocytes promotes glycolysis, while deletion of Hk1 in neonatal rat cardiomyocytes decreases glycolysis [133]. This experiment was undertaken in cultured rat cardiomyocytes, but the effect of this isoform switching in vivo has not been elucidated. Finally, a single cell (sc)RNA-seq experiment recently identified an embryonic protein of the electron transport chain, Cox8b, that is replaced around P0 by an adult isoform, Cox8a in ventricular cardiomyocytes [41]. In the case of all of these mitochondrial protein isoforms, the transcriptional mechanisms by which their expression is regulated are unclear and represent an interesting direction for future research.

## 4. Chromatin Remodeling and Epigenetic Control of Cardiomyocyte Maturation

Concurrent with the many biochemical, structural, and molecular changes underlying postnatal cardiomyocyte maturation are chromatin restructuring events that, as is the case with transcription factor activity, are not well characterized during the postnatal period. For example, Brg1, the ATPase subunit of the SWI/SNF chromatin remodeling complex, promotes proliferation in fetal mouse cardiomyocytes by activating BMP signaling and suppressing *Cdkn1c* [134] in conjunction with developmental cardiac transcription factors, such as Tbx5, Gata4, and Nkx2.5 [135]. In addition, Brg1 activates *Myh7* expression in fetal cardiomyocytes, and although its expression decreases in adult cardiomyocytes, it is induced following ischemic stress to promote pathological *Myh7* expression in adults [134]. The expression pattern of the histone acetyltransferase p300 is similar to that of Brg1. Interestingly, p300 activity in fetal cardiomyocytes promotes histone modification on numerous genes as a cofactor with Gata4, Nkx2.5, and Mef2C, but its adult expression levels remain low except following ischemic stress [136,137,138,139]. Finally, polycomb repressive complex 2 (Prc2) promotes a dynamic histone methylation pattern linked to fetal cardiomyocyte cell cycle activity [140]. Subsequently, the overexpression of Ezh1 (a subunit of Prc2) promotes cardiac proliferation and regeneration in P10 mice [141], suggesting that Prc2 is a major regulator of postnatal cardiomyocyte maturation. Given the importance of epigenetics and chromatin accessibility in regeneration, these modifications may explain why prenatal overexpression of some transcription factors promotes cardiomyocyte proliferation while induced expression of the same factor in adult cardiomyocytes promotes only hypertrophic growth. As such, these chromatin changes must be taken into consideration, rather than viewing individual transcription factors as independent units capable of inducing a response on their own. Although chromatin remodeling complexes and epigenetic changes exert large-scale effects on gene expression, understanding how they intersect with individual transcription factors in cardiomyocytes during the postnatal period may also elucidate the molecular mechanisms underlying seemingly contradictory functions during the maturation process.

Looking beyond transcriptional control, post-transcriptional regulation of genes by RNA binding proteins and miRNAs also have been implicated in postnatal cardiomyocyte maturation. Some of the major miRNAs in cardiac development and disease include miR-133, which promotes embryonic cardiomyocyte proliferation, miR-208, which is upregulated during heart failure to promote the pathological remodeling of cardiomyocytes, as well as miR-128 and miR-15, which have been implicated in postnatal cardiomyocyte cell cycle exit in mice [142]. While the targets of these miRNAs are not well-defined, they represent attractive candidates for promoting cardiomyocyte regeneration after cardiac injury. The CELF and MBNL families of RNA binding proteins regulate alternative splicing events during mouse cardiomyocyte development, with Celf1 and Celf2 expressed from embryonic stages until approximately P6 and P10, respectively, and Mbnl expression beginning at P5 and continuing to adulthood [143]. By regulating alternative splicing and mRNA degradation, Celf and Mbnl proteins compete for RNA targets and may contribute to post-translational regulation of differential gene expression that occurs during postnatal cardiomyocyte maturation [144]. However, overexpression of Celf1 in adult mouse cardiomyocytes causes dilated cardiomyopathy [145], demonstrating that this RNA binding protein alone is not sufficient to induce a fetal reversion phenotype and further demonstrating the need for understanding all hierarchical mechanisms that underly cardiomyocyte maturation. The major transcriptional regulators discussed are summarized in Table 1.

## 5. Conclusions

In mammals, birth induces many changes in cardiomyocytes, which must rapidly respond in order to support the needs of the growing heart and body. The increases in myocyte size and force generation by the sarcomeres comes at the expense of cell cycle exit, and represents a barrier to improving regenerative medicine after injury. While it is tempting to push adult cardiomyocytes to a regenerative fetal state, the robust activation of the fetal gene program, such as by overexpressing cyclins or YAP, may not be beneficial in the long term. This may be due to significant differences in the entire chromatin and transcriptional landscape in adult versus fetal cardiomyocytes; thus, targeting one individual gene is insufficient to overcome these changes. It is clear that simply forcing adult cardiomyocytes to take on fetal characteristics also does not take into account key aspects of cardiomyocyte biology, such as metabolic maturation and adult sarcomeric protein isoform expression, which themselves are not yet fully understood. Further, while a transient dose of these transcriptional regulators may be beneficial, most experimental models involve long-term overexpression or permanent deletion, which prevents de-differentiated cardiomyocytes from subsequently maturing and may contribute to the pathological phenotypes in the long term. By comprehensively investigating the numerous changes that occur during the postnatal period of cardiomyocyte maturation, and investigating use of transient expression models, we may begin to have a better grasp on specific ways to reverse the maturation process in terminally differentiated adult cardiomyocytes.

In addition to improving therapeutic strategies aimed at adult cardiomyocyte regeneration after injury, increasing our understanding of the transcriptional regulation of postnatal cardiomyocyte maturation may also contribute significantly to cardiac disease studies. Human induced pluripotent stem cell (hiPSC)-derived cardiomyocytes represent a promising tool for developmental studies and drug discovery, but are limited in usefulness by their inability to mature into an adult-like state [162]. Currently, the exact mechanisms that would promote in vitro maturation are unknown. By improving upon our understanding of regulatory mechanisms involved in postnatal cardiomyocyte maturation, we may be able to shed light on ways to recapitulate maturation in hiPSC cardiomyocytes. This advance would provide an invaluable tool to further our understanding of cardiomyocyte biology, disease and regeneration.

Most studies of cardiomyocyte regeneration compare embryonic or fetal cardiomyocytes, which are regenerative, against adult cardiomyocytes with limited regenerative capacity. With the numerous differences between these two cell states, there may not be a simple way to promote fetal characteristics in adult cardiomyocytes. The recent focus on an intermediate (postnatal) state may give us more informative clues about what targets and pathways must be exploited therapeutically. To date, however, studies specifically looking at the transcriptional regulation of postnatal cardiomyocytes have been limited. Transcriptional regulation changes during this period include major shifts in expression of downstream target genes critical for postnatal cardiac function that are likely influenced by transcription factor protein modifications or alterations of their protein binding partners. Further, these multifaceted regulatory mechanisms are in many cases poorly understood, and, while many interesting transcriptional signaling targets are studied for their role in cardiac development and maturation, there is still a gap in knowledge of the crosstalk between various pathways. A better grasp of these processes could have enormous implications for treatment and management of cardiovascular disease.

## Figures and Tables

**Table 1:** Transcriptional regulators of postnatal cardiomyocyte maturation, their targets, roles in embryonic and postnatal/adult cardiomyocyte function, and human heart defects associated with mutations/abnormalities in these genes.

Gene Name	Transcriptional Targets in Cardiomyocytes	Role in Cardiomyocyte Development	Role in Postnatal Cardiomyocyte Maturation	Associated Human Heart Defects
Btg2 [65]	Unknown	Unknown	Contributes to cell cycle exit	Unknown
E2f2/4 [70,71,72,146]	repressor of p53; retinoblastoma protein; activator of cyclins A, E, and D3 (unknown if direct or indirect)	Promotes proliferation	Downregulation contributes to cell cycle exit	Unknown
ErbB2/4 [53,54,55,56,147]	activates MAPK and AKT signaling cascades	Promotes proliferation and ventricular trabeculation	Downregulation contributes to cell cycle exit	Abnormalities associated with left ventricular outflow tract defects
ERRs [118,119,120,121,122,148,149,150]	activator of *Gata4*, succinate dehydrogenase genes, electron-transferring flavoproteins, and components of oxidative phosphorylation and the electron transport chain (including *Atp5g3*, *Coq7*, *Cox6c*, *Ndufa8*, *Ckmt2*, and *Slc25a4*)	Not expressed	Promotes mitochondrial oxidative metabolism	Downregulated in human heart failure; alterations are predictive for heart failure
FoxM1 [7,69]	Activator of *Igf1*; repressor of *p21*, *p27*	Promotes proliferation downstream of AKT	Downregulation contributes to cell cycle exit	Unknown
FoxO1/3 [69,77,131]	Repressor of *Igf1*; activator of *p21*, *p27*	Not activated	Promotes postnatal cell cycle exit; promotes survival	Unknown
Gata4 [19,56,68,74,87,88,90,91,102,109,135,140,151]	Activator of *Cdk2*, *Cdk4*, *Hand2*, *BNP*, *Myh6*; repressor of *Cdkn1c*	Promotes early differentiation and proliferation	Promotes hypertrophic growth, promotes expression of mature sarcomeric protein isoforms	Mutations associated with instances of congenital heart defects
Hand2 [74,75,152]	Unknown	Promotes proliferation in the developing outflow tract and left ventricle	Not expressed	Mutations associated with familial congenital heart defects
HIF-1α [123,153]	Repressor of *Mfn1*, *Mfn2*, *Opa1*	Maintains immature mitochondrial function in hypoxic environment	Downregulation promotes mitochondrial biogenesis, growth, and maturation	Elevated levels of protein in acyanotic congenital heart disease with hypoxemia
Isl1 [73,74,75,154]	Activator of *Fgf*s, *Bmp*s, *Hand2*	Promotes proliferation and heart field specification	Not expressed	Mutations associated with congenital heart defects
Maf [126,127,128]	Activators of ARE enhancers; *Gsta1*, *HO-1*	Not expressed	Antioxidant effects to handle increased ROS production	Unknown
Mef2 [101,102,103,104,105,106,107,108,155]	Activators of *Myh6*	Promotes myofibril stability and sarcomere organization	Promotes myofibril stability and sarcomere organization; promotes expression of mature sarcomeric protein isoforms	Mutations associated with familiar congenital heart defects
Meis1 [77,99]	Activator of *p15*, *p16*, *p21*	Not expressed	Promotes cell cycle exit and hypertrophic growth in combination with Hoxb13	Unknown
Nkx2.5 [90,91,110,156]	Activator of *BNP*; miR-1	Promotes early differentiation and proliferation	Promotes hypertrophic growth and sarcomere organization	Mutations frequently associated with congenital heart defects
Nrf2 [128,129,157]	Activator of *Nrf-*1, ARE enhancers; *Gsta1*, *HO-1*	Promotes mitochondrial biogenesis	Antioxidant effects to handle increased ROS production; rapidly degraded in non-stressed conditions	Abnormalities associated with heart failure progression
PGC1α [111,112,113,114,115,116,117,118,119,120,121,122,158]	Activator of ERRs, activator of succinate dehydrogenase genes, electron-transferring flavoproteins, and components of oxidative phosphorylation and the electron transport chain (including *Atp5g3*, *Coq7*, *Cox6c*, *Ndufa8*, *Ckmt2*, and *Slc25a4*)	Promotes mitochondrial biogenesis	Promotes fatty acid oxidation while inhibiting glycolysis, promotes antioxidant properties in stressed conditions	Mutations associated with congestive heart failure
PPARs [111,112,113,114,115,116,117,122,159]	Activator of ERRs, activator of succinate dehydrogenase genes, electron-transferring flavoproteins, and components of oxidative phosphorylation and the electron transport chain (including *Atp5g3*, *Coq7*, *Cox6c*, *Ndufa8*, *Ckmt2*, and *Slc25a4*)	Promotes mitochondrial biogenesis	Promotes fatty acid oxidation while inhibiting glycolysis, promotes antioxidant properties in stressed conditions	Mutations associated with ventricular septal defects
Tbx20 [60,61,62,63,64,65,66]	Activator of *Ccna2*, *Cdde*, *Mycn*, *Erbb2;* repressor of *Cdkn1a*, *Meis1*, *Btg2*	Promotes cell specification and proliferation	Downregulation promotes cell cycle exit; promotes sarcomere and myofibrillar organization	Mutations associated with common congenital heart defects
Tbx5 [57,58,59,103,160]	Activator of *Cdk2*, *Cdk4*, *Nppa*, *Gja5*, *Scn5a*, *Myh6*	Promotes heart chamber growth and proliferation	Promotes conduction and ion channel homeostasis	Mutations associated with multiple congenital heart defects, including Holt-Oram Syndrome
Yap1 [49,50,51,52,130,131,132,161]	Activator of *Smad*s, *Tcf4*, *Parkin;* Repressor of Wnt signaling	Promotes proliferation	Downregulation promotes cell cycle arrest; promotes oxidative phosphorylation and mitochondrial homeostasis; promotes antioxidant properties in stressed conditions	Reduced levels associated with ventricular septal defects
